# Supplementary material for: Comparative effects of two heat acclimation protocols consisting of high-intensity interval training in the heat on aerobic performance and thermoregulatory responses in exercising rats
Source: PLoS One. 2020 Feb 21;15(2):e0229335. doi: 10.1371/journal.pone.0229335 (PMC7034902; doi:10.1371/journal.pone.0229335)
Supplement: Attachment — Submitted filename: Plos One - Bittencourt et al. 2019 - Response to reviewers.docx [file pone.0229335.s009.docx]

**Response to reviewers - PONE-D-19-18686**

Dear editor and reviewers,

Thank you for the review of the manuscript entitled “Comparative effects of two heat acclimation protocols consisting of high-intensity interval training in the heat on aerobic performance and thermoregulatory responses in exercising rats”. We have carefully addressed all the major and minor comments from the reviewers and then modified the manuscript accordingly; changes made to the manuscript are highlighted in the revised version. We answered the questions in a point-by-point fashion, as presented on the next pages. We believe that the revised version of our manuscript has been greatly improved and hope that it will be suitable for publication.

P.S.: the page and line numbers indicated in the answers below correspond to the revised manuscript with track changes.

**Reviewer #1**

This manuscript describes a study into the effects of two high intensity interval training modalities on performance and thermoregulatory responses in rats in the heat. The manuscript is clear and well-written, and merit has to be given to the control of the study. However, it is difficult to ascertain whether the similarities/ differences observed between the two HIIT protocols are due to the variations in duration or intensity of exercise.

We thank the first reviewer for the constructive comments on our manuscript. Please see below our responses to these comments.

Major comment.

1- Changes in workload from pre to post interventions, although not statistically significant, appear to have a large effect between HIIT 100% and HIIT 85% (Figure 5). In performance studies, effect sizes are important as they provide a magnitude of difference, as opposed to a simple non-significance, which does not provide an extent of difference. Within this context, HIIT 85% may be practically better for performance based solely on Figure 5B. However, it is difficult to ascertain whether this effect was due to a longer duration (higher workload) in HIIT 85%, as the workloads between conditions were not matched (Figure 1B). As such, the current study design and results do not help to clarify the second hypothesis.

Thank you again for the very helpful comments. To address this major comment, we have made several changes to the manuscript, which are listed as follows:

1- we have calculated effect sizes for most of the comparisons made between the two HIIT protocols (please see how the calculation was made in page 15, lines 305 to 311). More specifically, effect size comparisons revealed that: 1- the training-induced increase in workload was greater in the HIIT_85%_ than in the HIIT_100%_ group (i.e., a moderate effect size, figure 5B); 2- the training-induced increase in thermoregulatory efficiency was also greater in the HIIT_85%_ than in the HIIT_100%_ group (i.e., a large effect size, figure 8B). This information was included in the revised manuscript: page 22, lines 457 to 460, and page 24, lines 517 to 520.

2- we have clarified our second hypothesis (page 6, lines 116 to 124). The revised hypothesis states that the improvement in aerobic performance was expected to be more evident after the HIIT_100%_, whereas thermoregulatory adaptations were expected to be more evident after the HIIT_85%_.

3- we have amended the conclusion (page 33, lines 711 to 724), which is now formulated based on the results generated by our experiments.

4- we have acknowledged in the revised discussion that future experiments should compare the adaptations induced by HIIT protocols consisting of training sessions at different intensities but with the same total workload (pages 28 and 29, lines 604 to 607).

Specific comments.

2- Line 531. Indeed, it is difficult to determine the degree of influence that intensity had on thermoregulatory responses in this study.

We agree with the comment made by the reviewer, because our method does not allow us to isolate the influence of intensity on the adaptations induced by heat acclimation. To avoid erroneous interpretation of our data, we have thoroughly revised the discussion section. For example, the revised manuscript highlights a possible role of exercise / heat exposure duration in the induction of heat acclimation (page 28, lines 598 to 607). Moreover, as stated earlier, we have acknowledged in the revised discussion that future experiments should compare the adaptations induced by HIIT protocols consisting of training sessions at different intensities but with the same total workload (pages 28 and 29, lines 604 to 607).

3- Line 612. The suggestion and the paragraph should be related to a specific result measured in the study.

The information written in the penultimate paragraph of the discussion does not relate to any specific result generated by the present study. However, we believe that this paragraph is important, because it helps to address a question mentioned earlier in the discussion: “The improved thermoregulatory efficiency in our rats has most likely resulted from adaptations in cutaneous heat loss and/or in metabolic heat production”. In addition, this paragraph indicates to readers a possible experiment that should be performed in the future (i.e., the evaluation of running economy in rats subjected to HIIT protocols in the heat). Therefore, we kindly ask the reviewer to allow us to maintain this paragraph in the revised manuscript.

4- Line 632. It is normally true that higher intensity interval bouts are more time efficient in achieving work done. In this study, despite a lesser exercise time in the HIIT 100% compared with HIIT 85%, HIIT 85% appears to have a large performance effect over HIIT 100%. Moreover, colonic and tail temperatures were not different. Therefore, it is difficult to confidently conclude that HIIT 100% was more efficient in inducing heat acclimation.

We agree with the important comment made by the reviewer. The statement that “that HIIT 100% was more efficient in inducing heat acclimation” was deleted to address the issues raised by both reviewers (please see the revised conclusion in page 33, lines 711 to 724). In addition, we have made a great effort to improve the discussion of our findings. The revised manuscript no longer states that a lower thermoregulatory efficiency means a greater thermoregulatory strain. Instead, we have tried to discuss our thermoregulation data (colonic and tail-skin temperatures and thermoregulatory efficiency) in an integrated manner.

**Reviewer #2:**

Major Points

1. Discussion: Clarity required.

I think the discussion may be complicated by temporal and workload (intensity and bout number) differences in the training design. However the incremental protocol offers the most controlled comparison of the heat acclimation with the different exercise training programs, Figure 5 demonstrated the 85% intensity training produces the greatest workload improvement (not significant) in the incremental test protocol and the greatest attenuation of heat relative to the distance travelled (ie workload). This indicated thermodynamic efficiency is improved post acclimation in this group, which appear to be contrary to the conclusion.

We thank the second reviewer for the constructive comments on our manuscript. Please see below our responses to these comments.

Regarding the data presented in figure 5, despite the lack of statistical differences between groups subjected to HIIT protocols, the change in workload induced by training was 51% greater in the HIIT_85%_ than in the HIIT_100%_ group. The greater value in the HIIT_85%_ group can be classified as a moderate effect size (ES = 0.74). This information was inserted in the results section of the revised manuscript (page 22, lines 457 to 460). Please note that we have inserted effect size assessment (i.e., Cohen’s *d*) in the revised manuscript, as suggested by the first reviewer.

Regarding the data presented in figure 8, we noticed a mistake in the figure that was originally submitted. The *post hoc* test (i.e., Tukey’s test) could not reveal a significant difference between the HIIT_85%_ and HIIT_100%_ groups (p = 0.100). We are sorry for this mistake, which was corrected in the revised version of figure 8.

Therefore, despite the lack of statistical differences between groups subjected to HIIT protocols, the change in the ratio induced by training was 165% greater in the HIIT_85%_ than in the HIIT_100%_ group (Figure 8B); the greater value in the HIIT_85%_ group can be classified as a large effect size (ES = 1.39). This information was inserted in the results section of the revised manuscript (page 24, lines 517 to 520).

We agree that the result mentioned in the paragraph above is contrary to our original conclusion. Therefore, we have amended the conclusion to properly address the present findings (page 33, lines 711 to 724).

2. Considering this is the most controlled performance measure between the groups. More needs to be made of this combination of results in the discussion. It may be that whilst intensity is effective, work volume may be a more effective factor in acclimation process.

We have made a great effort to improve the discussion of our findings. Of note, the revised manuscript no longer states that a lower thermoregulatory efficiency means a greater thermoregulatory strain. Instead, we have tried to discuss our thermoregulation data (colonic and tail-skin temperatures and thermoregulatory efficiency) in an integrated manner. For example, we have changed information in the following sentences / paragraphs: page 26, lines 537 to 547; page 27, lines 566 to 572; pages 28 and 29, lines 583 to 607.

3. Furthermore, clarity of the sentence beginning line 630 needs to be revisited. In particular, what is meant by more efficient strategy? when the results were not entirely comparable in terms of thermal changes as demonstrated in Fig 8 B

The statement that “that HIIT 100% was more efficient in inducing heat acclimation” was deleted to address the issues raised by both reviewers. We agree that information in this sentence was contradictory to data presented in figures 5 and 8. The revised conclusion is now formulated based on the results generated by our experiments (page 33, lines 711 to 724).

Minor Points

1. Abstract. Line 29, "exercise at" should read "exercise to fatigue at"

The sentence was changed as follows (page 2, line 29): “Twenty-three adult male Wistar rats were initially subjected to an incremental-speed exercise at 32°C until they were fatigued and then …”.

2. Abstract. Line 40, when compared to UN rats. I think that this would be better described "when compared to pre-intervention test". Using UN both within the brackets and the subsequent statement is confusing.

The abstract was revised to present the workload and ratio values calculated from the second incremental-speed exercise that was performed after the interventions. Therefore, the sentence was changed as follows (page 2, lines 36 to 42): “After the intervention period, rats subjected to both HIIT protocols attained greater workloads (HIIT_100%_: 313.7 ± 21.9 J vs. HIIT_85%_: 318.1 ± 32.6 J vs. UN: 250.8 ± 32.4 J; p < 0.05) and presented a lower ratio between the change in T_COL_ and the distance travelled (HIIT_100%_: 4.95 ± 0.42 °C/min vs. HIIT_85%_: 4.33 ± 0.59 °C/min vs. UN: 6.14 ± 1.03 °C/min; p < 0.001) when compared to UN rats.” We hope that this change has improved clarity of the abstract. Moreover, please note that the abstract was further edited to reduce its size, thereby not exceeding the 300-word limit.

3. Introduction. Line 105-108

The second hypothesis, regarding comparisons between HIIT protocols. I do not understand the logic of the hypothesis based on the higher intensity creating a greater thermoregulatory strain. I think that that the total workload over a session would better reflect the total endogenous heat generation and thus with the lower intensity protocol design in this study seemingly performing more work over a training session.

Thank you for the very good comment. The text in the introduction was changed to explain the effects of exercise intensity and duration on thermoregulatory responses, particularly under conditions of uncompensable heat stress (pages 5 and 6, lines 95 to 102). Moreover, the second hypothesis was revised: it now states that the improvement in aerobic performance was expected to be more evident after the HIIT_100%_, whereas thermoregulatory adaptations were expected to be more evident after the HIIT_85%_ (page 6, lines 116 to 124).

4. Procedures. lines 176-178.

I am a little confused by the Smax formula which doesnt make sense to me. Smax = S + (t/180). S is in units of m.min-1 yet the (t/180) which is in a time unit is added to it. How does the addition of these two factors reconcile to result in a unit of velocity for Smax. I know it is referenced but I dont have access to that article to check the original source.

Thank for the suggestion. The equation was amended to improve its clarity. The amended equation is presented as follows (page 12, lines 238 to 242): S_MAX_ = S + (t_1_ / t_2_), where S = speed in the last completed stage in m/min; and t_1_ = time spent in the incomplete stage in seconds; and t_2_ = duration of each stage, which corresponds to 180 seconds.

Therefore, t_1_ and t_2_ are both expressed in seconds. Thus, the second factor of the equation corresponds to seconds divided by seconds and, therefore, has no unit.

5. Results. Figure 4.

This is important in representing thermal load. However, I believe the units of the ratio axis in parentheses is wrong. It should be per m, not per min.

The reviewer is correct. We are sorry for this mistake. The units inside the parentheses were corrected in figure 4.

6. Results. Figure 6. again the units. per m? not per min?

If this comment is related to figure 8, the reviewer is correct again. Again, we are sorry for this mistake. The units inside the parentheses were corrected in figure 8.
