# Supplementary material for: Comparative effects of two heat acclimation protocols consisting of high-intensity interval training in the heat on aerobic performance and thermoregulatory responses in exercising rats
Source: PLoS One. 2020 Feb 21;15(2):e0229335. doi: 10.1371/journal.pone.0229335 (PMC7034902; doi:10.1371/journal.pone.0229335)
Supplement: Attachment — Submitted filename: Plos One - MAG et al. - Second response to reviewers.docx [file pone.0229335.s010.docx]

**Response to reviewers - PONE-D-19-18686R1**

Dear editor and reviewers,

Thank you for the review of the manuscript entitled “Comparative effects of two heat acclimation protocols consisting of high-intensity interval training in the heat on aerobic performance and thermoregulatory responses in exercising rats”. We have carefully addressed the comment from the second reviewer and then modified the manuscript accordingly; changes made to the manuscript are highlighted in the revised version with track changes. We believe that the revised version of our manuscript has been improved and hope that it will be suitable for publication.

P.S.: the page and line numbers indicated in the answer below correspond to the revised manuscript with track changes.

**Reviewer #1**

Thank you for thoroughly addressing comments suggested. Please ensure data is uploaded and available.

We thank the first reviewer for the positive comment. Moreover, we confirm that all data were uploaded in Microsoft Excel spreadsheet format and are available for readers.

**Reviewer #2:**

I believe the revision has tightened the manuscript. However, considering the rectification of the error, I believe that now the importance of non HIIT protocols becomes more relevant. The endothermic load of exercise may be all that is needed for acclimation, constant workload of HIT protocols (any protocol). It may just be a question of exposure to elevated temperature for long enough period of time to stimulate adaptations. This is not an insignificant point. I think a section addressing this point in the discussion may be warranted as there is no difference between these protocols.

We thank the second reviewer for the constructive comment. A paragraph was inserted in the discussion (page 29, lines 595-607) to address his/her comment. This paragraphs highlights that: a- the endothermic heat production during exercise and the resulting body heat storage may drive acclimation in rats; b- thermoregulatory adaptations likely result from different combinations among environmental heat stress and exercise intensity and duration; c- depending on how training sessions are planned, non HIIT protocols (e.g., moderate-intensity continuous training) can be effective in inducing heat acclimation in rats.
